# Editorial: Advancing biomechanics: enhancing sports performance, mitigating injury risks, and optimizing athlete rehabilitation, volume II

**DOI:** 10.3389/fspor.2026.1872907

**Published:** 2026-06-02

**Authors:** Lamia Ben Ezzdine, Wissem Dhahbi, Laurence Chèze

**Affiliations:** 1 High Institute of Sport and Physical Education of Ksar Said, University of Manouba, Manouba, Tunisia; 2 Research Unit “Sport Sciences, Health and Movement”, High Institute of Sports and Physical Education of Kef, University of Jendouba, Kef, Tunisia; 3 Training Department, Police College, Qatar Police Academy, Doha, Qatar; 4 Univ Lyon, Université Claude Bernard Lyon 1, Univ Gustave Eiffel, LBMC UMR_T9406, Lyon, France

**Keywords:** electromyography, foot strike pattern, footwear biomechanics, ground reaction force, injury prevention, lower limb biomechanics, motion capture, musculoskeletal modeling

## Introduction

The second volume of this Research Topic extends the empirical and methodological scope established in Volume I, assembling 15 contributions that address three convergent imperatives in contemporary applied biomechanics: optimizing the mechanical efficiency of athletic footwear and locomotion, advancing sport-specific neuromuscular diagnostics and training, and quantifying injury-risk mechanisms across diverse movement contexts. The collection spans original investigations, a systematic review with meta-analysis, narrative and scoping reviews, and a case report, representing 77 authors and generating more than 49,000 combined views and downloads at time of publication. The instrumentation represented across these articles, from multi-camera optical capture systems and force platforms to surface electromyography (EMG), musculoskeletal (MSK) modeling, and machine learning (ML) classifiers, reflects the accelerating transition of biomechanical inquiry from controlled laboratory settings toward ecologically valid, data-intensive field applications.

## Footwear technology and foot strike biomechanics

Five contributions examine the mechanical interface between footwear design, foot strike pattern, and lower-limb loading, with direct implications for running performance and overuse injury prevention. Horiguchi et al. investigated the independent and interactive effects of insole material (EVA vs. Sorbothane) and foot strike pattern on shock attenuation during constant-speed running in nine male participants. Peak and loading rate of the vertical ground reaction force (GRF) were higher in the rearfoot strike condition regardless of insole type, and Sorbothane reduced the GRF loading rate selectively under rearfoot conditions; the foot strike pattern thus exerted a stronger influence on impact characteristics than insole material. Extending the analysis to category-defining contemporary footwear, Giachetti Martin et al. conducted a PRISMA-compliant systematic review and meta-analysis of 15 crossover trials comparing carbon-plated with non-carbon-plated shoes in healthy adults. Random-effects modeling yielded no statistically significant group differences in leg stiffness, knee power, hip power, or metatarsophalangeal (MTP) joint power, with only a borderline reduction in ankle power (SMD = −0.71; 95% CI: −1.42 to 0.00; *p* = 0.05); certainty of evidence ranged from low to moderate, indicating that mechanical adaptations to carbon-plated footwear are more distal and subtle than commonly assumed. Yang et al. refined this question by testing three levels of longitudinal bending stiffness (LBS) in carbon-plated shoes on 15 elite male marathon runners. Medium LBS (0.40 Nm/deg) increased positive ankle joint work relative to low LBS (0.71 vs. 0.66 J/kg; *p* = 0.047) and reduced MTP dorsiflexion without elevating hip extension demands, establishing medium LBS as a biomechanically balanced design target for elite distance footwear. The role of experience-level and footwear interactions was addressed by Li et al., who compared patellofemoral joint (PFJ) and Achilles tendon (AT) loading in novice and experienced male runners across conventional and minimalist shoes under both rearfoot strike (RFS) and forefoot strike (FFS) conditions. PFJ contact force and AT impulse were consistently higher in novice runners irrespective of footwear or foot strike pattern; across experience levels, FFS reduced PFJ stress while elevating AT force and impulse relative to RFS, underscoring the necessity of graduated foot strike transitions to avoid load redistribution injuries in less-trained populations. Huang et al. investigated a related biomechanical trade-off during a stop-jump task, demonstrating that FFS produced a more posteriorly inclined GRF vector and a shorter stance time (396.75 vs. 433.48 ms; *p* = 0.01) without reducing jump height, while simultaneously increasing ankle internal rotation; this pattern suggests a trade-off between anterior cruciate ligament (ACL) protection and lateral ankle sprain risk that must be weighed explicitly when prescribing landing-technique training.

## Sport-specific performance and neuromuscular optimization

Five studies address sport-specific movement mechanics and the neuromuscular adaptations produced by targeted training interventions, with three contributions focusing on badminton and two on broader athletic contexts. Prończuk et al. evaluated a four-week EMG-guided stretch-shortening cycle (SSC) training program in 24 national-level badminton athletes, reporting reductions in EMG latency of 12.2–16.5 ms and an increase in reactive strength index (RSI) of 13.4% (*p* = 0.014) in the experimental group relative to a sham-feedback control. Principal Component Analysis and Linear Discriminant Analysis identified neuromuscular timing as the principal axis of group differentiation, while Random Forest and Multilayer Perceptron classifiers predicted post-intervention responder status with an area under the curve (AUC) of 0.92, demonstrating the utility of ML-based approaches for individualized diagnostics despite the constraints of small-sample designs. Tajik et al. applied non-negative matrix factorization (NMF) to EMG signals from 15 shoulder muscles in 20 elite badminton players executing the forehand overhead smash and validated the resulting synergy structures against OpenSim MSK simulation outputs. Three synergies accounted for more than 90% of variance in both experimental and modeled data, with strong weight vector agreement (W1: 0.81, W2: 0.87, W3: 0.88) and activation coefficient correspondence (C1: 0.95, C2: 0.98, C3: 0.98), confirming the combined experimental-computational framework as a valid tool for analyzing high-velocity overhead movements. Xie et al. compared eight-week resistance training (RT) and complex training (CT) programs on lower-limb kinematics and kinetics during the badminton backhand forward lunge in 20 male amateur players, using Statistical Parametric Mapping (SPM1D) for continuous variable analysis. CT reduced both braking and recovery phase times (*p* = 0.002 and *p* < 0.01, respectively), while RT improved execution efficiency alongside reductions in knee and ankle loading, revealing complementary injury-risk and performance profiles that have practical implications for periodized training prescription. Aoyama et al. contributed a case report on five weeks of graded task-oriented throwing training in a 21-year-old baseball player with a seven-year history of the yips; post-intervention, subjective symptom frequency decreased by approximately one-third, and three-dimensional motion capture confirmed reduced variability in ball release angle and shoulder internal rotation at release, providing preliminary evidence for low-risk physical therapy as a viable management approach when pharmacological intervention is contraindicated or undesired. In the multi-species context of equestrian sport, Balog et al. synthesized 17 peer-reviewed studies through a narrative review, establishing that correct pelvic orientation, dynamic trunk control, and symmetrical weight distribution in riders are consistently associated with improved saddle pressure distribution and equine gait quality, while rider asymmetries, whether inherent or acquired, degrade dyadic performance even when the rider is unaware of the imbalance.

## Injury risk quantification and prevention strategies

The final cluster quantifies injury-relevant biomechanical variables and evaluates preventive or corrective interventions across team and individual sports. Mohr and De Santis reported that an eight-week constraints-led training program based on a 1-on-1 laser tag game produced a statistically significant reduction in peak knee abduction moment (pKAM) during a 135° change-of-direction (COD) task (*p* = 0.038; Cohen's d = 0.63) while simultaneously reducing COD completion time (*p* < 0.001; d = 2.47), resolving the recurrent conflict between ACL injury prevention and COD performance that has constrained traditional technique-instruction paradigms. Wang et al. quantified interlimb COD asymmetry in 42 college basketball players using the change-of-direction shuffle (CoDS) and 505 tests, finding statistically significant dominant-to-nondominant differences in both tasks (asymmetry scores of 3.66 and 2.54; *p* < 0.01 for each), yet poor-to-slight Kappa coefficient agreement across tests (range: −0.07 to 0.22), establishing that lateral and forward-backward COD asymmetries are functionally dissociable and require task-specific assessment rather than a single composite metric. Tilp et al. surveyed 175 competitive volleyball players across six countries on the association between spike technique and shoulder injury, finding no statistically significant difference in injury or symptom frequency between bow-and-arrow and circular techniques in chi-square analyses (*p* > 0.05 for all comparisons), although descriptive trends suggested potentially higher rates with bow-and-arrow technique in female players, a gender-specific signal that warrants prospective investigation with objective biomechanical measurement. van Trigt et al. addressed an unresolved equipment-safety question in rowing by comparing erector spinae EMG activation during 500-m on-water rowing with 0-degree and 5-degree angled oar blades across 1,443 analyzed strokes in seven collegiate rowers; SPM analysis identified no significant activation differences, providing initial evidence that the modified blade configuration does not elevate lumbar injury risk during acute exposure. Pavlasová et al. concluded the collection with a scoping review mapping landing biomechanics methodology in gymnastics across eight studies and 212 participants aged 8 to 25 years, identifying superior neuromuscular control in gymnasts relative to untrained individuals, alongside pronounced methodological heterogeneity in stabilization assessment and the complete absence of studies examining concurrent relationships among muscle strength, EMG activation, and stabilization metrics; standardized protocols represent the most pressing methodological requirement for progress in this area.

## Synthesis and outlook

The 15 contributions assembled in this volume collectively demonstrate both the disciplinary maturity and the persisting methodological gaps of applied biomechanics in sport. The convergence of optical motion capture, force measurement, EMG, MSK modeling, and ML-based analytics is progressively enabling individualized, ecologically valid assessments of athletic performance and injury risk. Several articles identify specific priorities for the field: prospective longitudinal designs linking acute biomechanical variables to clinical injury incidence, standardized assessment protocols in gymnastics and equestrian sport, more balanced sex representation and larger samples in footwear research, and the interaction between LBS gradations and individual runner morphology across race distances. The editors thank all contributing authors, the review editors, and the editorial staff of Frontiers in Sports and Active Living for the thoroughness and commitment that shaped this collection.

